# In search of a one plan solution for VMAT post‐mastectomy chest wall irradiation

**DOI:** 10.1002/acm2.12948

**Published:** 2020-06-27

**Authors:** T. T. Monajemi, P. A. K. Oliver, A. Day, M. Yewondwossen

**Affiliations:** ^1^ Department of Radiation Oncology Dalhousie University Halifax Nova Scotia Canada; ^2^ Department of Physics and Atmospheric Science Dalhousie University Halifax Nova Scotia Canada

**Keywords:** bolus, brass mesh, chest wall radiation therapy, skin dose, tangents, VMAT

## Abstract

**Purpose:**

This study was designed to evaluate skin dose in both VMAT and tangent treatment deliveries for the purpose of identifying suitable bolus use protocols that should produce similar superficial doses.

**Methods:**

Phantom measurements were used to investigate skin dose in chest wall radiotherapy with and without bolus for 3D and rotational treatment techniques. Optically stimulated luminescence dosimeters (OSLDs) with and without housing and EBT3 film were used. Superflab (3, 5, and 10 mm) and brass mesh were considered. Measured doses were compared with predictions by the Eclipse treatment planning system. Patient measurements were also performed and the bolusing effect of hospital gowns and blankets were highlighted. The effect of flash for VMAT plans was considered experimentally by using 2 mm couch shifts.

**Results:**

For tangents, average skin doses without bolus were 0.64 (EBT3), 0.62 (bare OSLD), 0.77 (jacketed OSLD), and 0.68 (Eclipse) as a fraction of prescription. For VMAT, doses without bolus were 0.53 (EBT3), 0.53 (bare OSLD), 0.64 (jacketed OSLD), and 0.60 (Eclipse). For tangents, the average doses with different boluses as measured by EBT3 were 0.99 (brass mesh), 1.02 (3 mm), 1.03 (5 mm), and 1.07 (10 mm). For VMAT with bolus, average doses as measured by EBT3 were 0.83 (brass), 0.96 (3 mm), 1.03 (5 mm), and 1.04 (10 mm). Eclipse doses agreed with measurements to within 5% of measurements for all Superflab thicknesses and within 15% of measurements for no bolus. The presence of a hospital gown and blanket had a bolusing effect that increased the surface dose by approximately 10%.

**Conclusions:**

Results of this work allow for consideration of different bolus thicknesses, materials, and usage schedules based on desired skin dose and choice of either tangents or an arc beam techniques.

## 1 INTRODUCTION

Post‐mastectomy radiotherapy can pose significant treatment planning challenges. Traditional three‐dimensional (3D) conformal treatment planning options, including those with an electron patch, sometimes result in large dose inhomogeneity, inadequate target coverage, or excessive ipsilateral lung and heart doses. Even though the low‐dose wash of volumetric modulated arc therapy (VMAT) generally excludes it as the first option,^1^ VMAT planning techniques have gained some traction in recent years for treating difficult‐to‐plan post‐mastectomy and intact breast cases.^2^, ^3^, ^4^, ^5^ The primary advantage of VMAT over 3D techniques is sparing of heart and ipsilateral lung from high doses without sacrificing target coverage. This advantage comes at the price of higher dose to contralateral breast and lung and even higher low dose to ipsilateral organs at risk.

Differences in dosimetry between VMAT and tangential treatments are largely the result of the differences in incident angles of the beam. In tangential treatments, the beams are incident from two angles, medial and lateral. In VMAT planning, the planner may choose to limit more *en face* components of the beam by restricting the ranges of arc angles^6^ or they may choose to use an arc extending from medial to lateral angles and let the optimization objectives drive the weighting of tangential versus *en face* components.^2^ Regardless of the planning technique, there will be more *en face* weighting of the incident beam angles in VMAT compared with 3D conformal tangents.

The presence of the *en face* component raises questions regarding skin dose (including the role of bolus) and the importance of flash in VMAT planning. Historical treatments involving tangential fields with wedges facilitated an intuitive appreciation for how flash conferred reduced sensitivity to dose variation related to breathing motion, swelling, and setup uncertainty. Since forward planned IMRT effectively reproduces wedged tangents behavior (to a first approximation), similar understanding for field‐in‐field treatments exists. The variable gantry angle delivery and aperture modulation that characterizes VMAT treatment deliveries, however, makes prediction of the effects of flash more difficult. These changes in treatment characteristics also make it difficult to assume that skin dose either without or under a bolus would be the same for the two treatment techniques, potentially due to variability in path length through the skin and/or bolus as a function of gantry angle. To complicate matters further, a consensus definition of skin is difficult to establish. The ICRU states that the superficial layers of interest include the dermal lymphatics (to a depth of ~1 mm) and the basal cell layer at about 70 microns.^7^ Practical dosimetric quantities extracted from treatment planning systems are often on the order of 2 mm in thickness.^8^ For our purposes, we define herein the dose reported from *in vivo* dosimeters as representative of skin dose and we evaluate their behavior under different irradiation conditions.

Regardless of planning technique, to date, there has been a lack of consensus on whether the routine use of bolus in post‐mastectomy radiation therapy is necessary or not.^9^, ^10^ The guidelines of the American Society of Clinical Oncology first published in 2001^11^ and later updated in 2016^12^ stated that “*whether it is necessary to apply the bolus every day, less frequently, or at all is uncertain*.” As such, whether bolus is used routinely or not, its thickness and frequency are often decided by clinical experience and vary from center to center.^13^ Regardless of whether bolus is used or not at a clinic, its thickness and frequency, when using a VMAT technique it might be desirable to match the skin dose to that which is consistent with clinical practice as established by the three‐dimensional technique at that center.

To date, only a limited number of studies have investigated skin dose in VMAT treatments of chest wall.^14^ Absent from the literature is a systematic study of the impact of different types and thicknesses of bolus on skin dose for both 3D conformal and VMAT treatment techniques. This study was designed to evaluate skin dose in both VMAT and tangent treatment deliveries for the purpose of identifying suitable bolus use protocols that should produce similar superficial doses. Skin dose in this setting is evaluated with three *in* *vivo* dosimetry measurements: Gafchromic film, optically stimulated luminescence dosimeters in their jackets, and OSLDs without their jackets. The second goal was to evaluate the effects of flash on dose variation caused by breathing motion in VMAT post‐mastectomy radiation therapy. Here the goal was to determine whether the implementation of flash was necessary or not since the presence of more *en face* beams might make the distribution less susceptible to changes in outer body contour position due to breathing.

## 2 MATERIAL AND METHODS

### 2.A Phantom

**Fig. 1:**
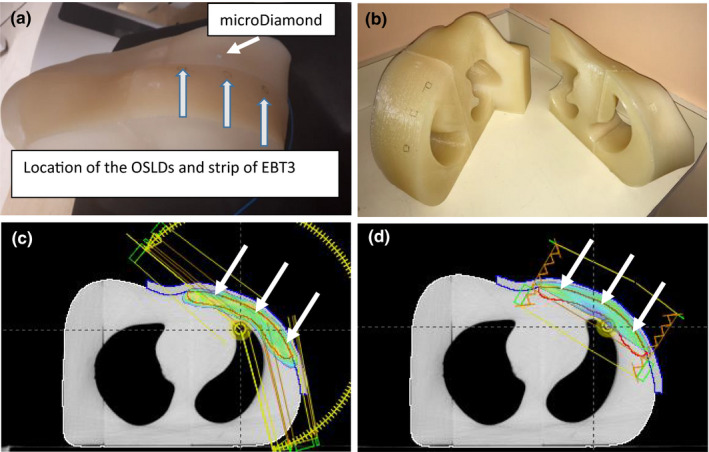
Picture of the three‐dimensional (3D) printed phantom used in this study. Location of the detectors is shown (a). The phantom consists of two segments for ease of handling (b). VMAT plan with 1 cm bolus, 95% dose wash is shown. The arrows point to the location of OSLDs and strip of film. CTV is shown (c). Tangent plan with 1 cm bolus, 95% dose wash is shown. The arrows point to the location of OSLDs and strip of film. CTV is shown (d).

A replica of a left‐sided chest wall CT set of a patient was 3D printed using PLA and a Lulzbot Taz5 MOARstruder, 100% infill (Fig. 1). The scan of the phantom showed a physical density of 1.1 g/cm^3^ and Hounsfield unit of 160. The phantom contains an insert to hold a PTW‐60019 microDiamond (PTW_Freiburg) detector. The microDiamond detector was chosen due to its shallow effective point of measurement (1 mm) and angular independence.^15^ This detector was only used for relative measurements and assurance that the phantom was set up reproducibly each time a measurement was repeated. No absolute dose readings were acquired using this detector.

### 2.B Planning

A CT image of the 3D‐printed phantom was taken with 2.5 mm slice spacing. Target and organ at risk volumes were drawn to permit creation of VMAT plans. A list of plans that were created by one experienced planner (Eclipse 13.6, AAA, 2 mm calculation grid) is given below. An identifier for each plan is provided using the following format: Technique:#Bolus:(±)#Cropping. Technique refers to VMAT or field in field tangents. #Bolus Refers to the bolus thickness in mm and #Cropping describes the modification distance from the outer body contour for the target evaluation structure (PTV_Eval). A positive value (+) indicates an expansion of the volume into the bolus (for the purpose of creating flash). A negative value (−) indicates a cropping from the outer body contour. It should be noted that while a PTV_Eval structure was present in all plans, it was only used to help shape the plans (through optimization) in the VMAT setting.

Eclipse treatment plans:

1. Field in field tangents:
  - No bolus: (Tangent:0Bolus:−3 mmCrop)
  - 5 mm synthetic bolus (i.e., bolus added in Eclipse using the Eclipse bolus functionality): (Tangent :5 mmBolus:–3 mmCrop)
  - 10 mm synthetic bolus: (Tangent:10 mmBolus:−3 mmCrop)
2. VMAT:
  - No bolus, PTV_Eval created by cropping the PTV structure 3 mm back from the outer body contour: (VMAT:0Bolus:−3 mmCrop)
  - 10 mm synthetic bolus, PTV_Eval cropped 0 mm from outer body contour (VMAT:10 mmBolus:0Crop)
  - 10 mm synthetic bolus, PTV_Eval expanded 5 mm into the bolus: (VMAT:10 mmBolus:+5 mmCrop)

A dose of 4000 cGy in 15 fractions was prescribed for all plans and only 6 MV photons were used. The planning aims are shown in Table 1. Although the prescription is to a point in tangents and a volume in VMAT, both techniques follow the PTV_EVAL coverage goals listed in Table 1. The VMAT plans included three arcs, the stop and start angles are shorter by 10° medially (310° for VMAT) and wider by 40° laterally (165° for VMAT) compared with tangential fields. Collimator angles are 20°, 340°, and 355°. The x jaw setting was roughly 16 cm for all three arcs and jaw tracking was used.

**Table 1:** Planning objectives used in this study.

PTV_Eval	V95% > 95% D_10cc_ < 4280 cGy D_0.03cc_ < 4400 cGy
Lung_Ipsi	V500 cGy < 60% V1000 cGy < 45% V2000 cGy < 25% Mean < 1000 cGy
Lungs_Total	V2000 cGy < 10% V750 cGy < 25% V500 cGy < 50% Mean < 600 cGy
Lung_Contra	V500 cGy < 10% Mean < 300 cGy
Heart	Mean < 300 cGy
Breast_Contra	V1000 cGy < 15% Mean < 400 cGy
Esophagus	D_0.03cc_ < 4000 cGy

### 2.C Phantom doses

All measurements were performed on a TrueBeam^TM^ linear accelerator. Cone beam CT images were acquired to ensure accuracy of phantom setup. Skin doses were evaluated with Gafchromic film (Ashland Advanced Materials, Bridgewater, USA) and nanoDot™ OSLDs (Landauer Inc., Glenwood, USA). OSLD measurements were performed with the dosimeter in its housing and with the dosimeter extracted from its housing (to minimize the effective depth of measurement compared to film^16^, ^17^). Data are clearly labeled with respect to the OSLD configuration in the subsequent sections.

To quantify representative skin doses, for each plan and bolus combination, we placed a strip of GafChromic EBT3 film of size 9 × 4 cm^2^ on the phantom [Fig. 1(a)] such that the longer side extended in the medial to lateral direction. The strip of film was then taken off and replaced with three OSLDs as indicated in Figure 1 and the plan was delivered twice more, once with three OSLDs in their housing and once with three OSLDs taken out of their housing. Care was taken to keep the room dark, not touch the OSLD, and place them back in their housing as soon as the treatment was delivered.

Scanned EBT3 images were measured using Film QA Pro software (Ashland Advanced Materials, Bridgewater, USA). The methodology described in Ref. [^18^] was used to analyze the film readings. OSLDs were read with an InLight microStar reader (Landauer Inc., Glenwood, USA). The effects of supralinearity (2.5‐3%) were corrected for OSLD readings of doses larger than 250 cGy.^19^ Dose‐dependent supralinearity correction factors were found by exposing a set of OSLDs to known doses in the range of 100–300 cGy.

Bolus materials considered were Superflabs of thickness 3, 5, and 10 mm and brass mesh (Whiting & Davis, Attleboro Falls, USA). Tangent:0Bolus: −3 mmCrop was delivered three separate times, once each with no bolus, with 3 mm Superflab, and with brass mesh. Tangent:5 mmBolus: −3 mmCrop and Tangent: 10 mmBolus: −3 mmCrop were delivered with 5 and 10 mm of Superflab, respectively. In addition, skin dose for 5mm Superflab QOD (every other day) fractionation was quantified by considering eight fractions of Tangents:0Bolus:‐3mmCrop and seven fractions of Tangent:5 mmBolus:−3 mmCrop.

VMAT: 0Bolus: −3 mmCrop, VMAT:10 mmBolus:0Crop, and VMAT:10 mmBolus: +5 mmCrop were delivered with no bolus following CBCT guided setup. To assess the effect of small breathing motion on skin dose, a vertical table shift of 2 mm (up) was introduced. The three VMAT plans were delivered again. The reading of the diamond detector was recorded for each plan with and without the table shift. As a result of the preliminary step of studying flash, we rejected plan VMAT:0Bolus: −3 mmCrop and VMAT: 10 mmBolus:0Crop (see results) and only focused on VMAT:10mmBolus: +5 mmCrop. Skin doses were measured with no bolus, brass mesh, and 3, 5, and 10 mm Superflab. If a patient is to receive the optimized VMAT:10mmBolus:+5mmCrop without bolus then the plan needs to be renormalized to adjust the monitor units down by ~5% to compensate for lack of attenuation in bolus. Similarly the monitor units need to be adjusted down by roughly 2.5% if the plan is to be delivered with 5 mm Superflab. Therefore, we adjusted the monitor units down by 5% (no bolus, 3 mm Superflab, and brass mesh), 2.5% (5 mm Superflab), and none (10 mm Superflab) when VMAT: 10 mmBolus: +5 mmCrop was delivered. In addition, skin dose for 5 mm Superflab QOD fractionation was quantified by considering eight fractions of VMAT: 10 mmBolus: +5 mmCrop (delivered with no bolus; MUs adjusted down by 5%) and seven fractions of VMAT: 10 mmBolus: +5 mmCrop (delivered with 5 mm bolus; MUs adjusted down by 2.5%).The effect of the monitor unit adjustment was to keep the mean dose to PTV (cropped 3 mm from the outer body contour) the same for all plans. Coverage metrics were not significantly affected by the scaling procedure. An alternative approach would be to create completely independent plans for bolus and no bolus fractions. However, this would require two separate full optimizations. We found that independent optimizations of bolus and no bolus plans did not improve plan quality. We adopted the approach of single optimization and renormalization as described here in the interest of clinical efficiency.

### 2.D Patient data

We performed *in vivo* readings on 10 QOD clinical VMAT: 10 mmBolus:0mmCrop VMAT patients with OSLDs in their housing on bolus (10 mm) and no bolus days. The OSLDs were placed on patient skin in a manner similar to Fig. 1(a). Similar to the phantom study on no bolus days, the patients received the same optimized plan but with the monitor units adjusted down by 5%. Since the majority of the patients at our institute use a gown and a hospital blanket, we repeated our phantom study in the presence of a hospital gown and knitted blanket. This was done to establish if the presence of these materials acts as a bolus and affects the baseline doses established by the phantom.

## 3 RESULTS

All skin doses are reported as a fraction of prescription dose, that is, skin dose of 0.6 means detector reading for one fraction is equal to 60% of 266.7 cGy which is the prescribed daily dose.

### 3.A Tangents

**Fig. 2:**
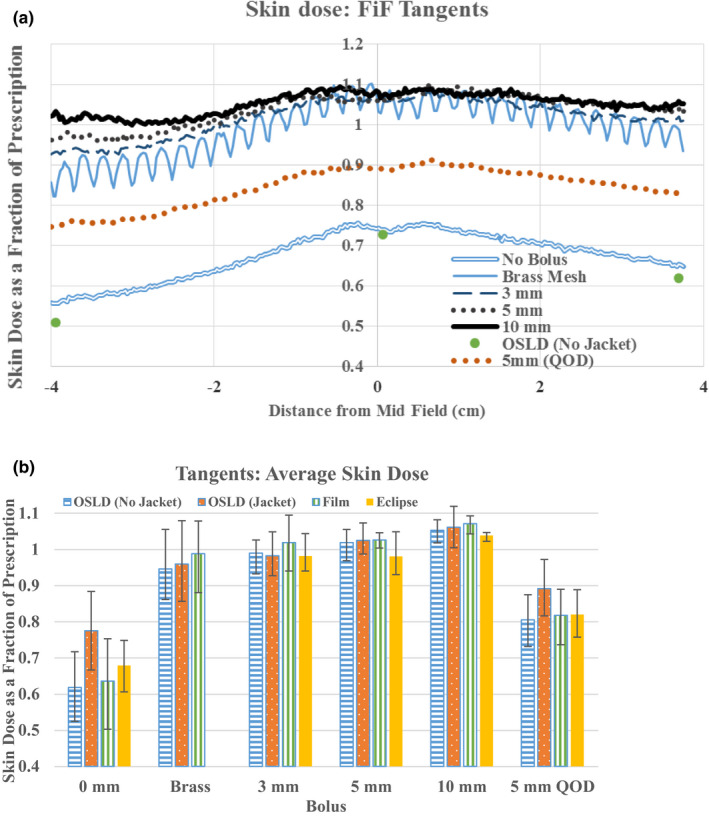
Three‐dimensional field in field superficial doses. Skin dose measured with EBT3 where negative distance is in the lateral direction. For comparison skin doses measured by bare OSLDs are shown for the case of no bolus (a). Average skin doses in the three OLSD locations as measured by jacketed, bare OSLD, EBT3, and calculated by Eclipse (b). All doses are expressed as a fraction of prescription dose. The bars indicated the range of measurements.

Skin doses as measured by EBT3 for the 3D FinF tangent plan are shown in Fig. 2(a). For comparison, dose measured with the bare OSLDs for the case of no bolus is shown on the same figure. The dose across the film was not uniform for any of the deliveries (in the medial–lateral direction). The highest doses were found in the middle of the film where the separation was smallest and decreased in both the medial and lateral directions. Interestingly, the decreases were asymmetric, with lower doses observed on the lateral side than the medial side. This may have resulted from a relative increase in the degree of *en face* directionality at the lateral side of the phantom, resulting in greater skin sparing at this side.

Doses measured by EBT3, bare OSLDs, jacketed OSLDs, and Eclipse for each bolus are shown in Fig. 2(b). Data points represent the average across the three measurement locations. The reported film doses were determined by calculating the average value of doses in 5 × 5 mm^2^ areas at locations corresponding to the OSLD locations. Eclipse doses were evaluated by creating regions of interest at the corresponding OSLD locations, with dimensions approximating the size of OSLDs with a nominal thickness of 1 mm. The reported values are the average of the mean value of the dose to each region of interest. The average skin dose as measured by the three detectors shows the largest variability for the case of no bolus: 0.64 (range: 0.54–0.73, EBT3), 0.62 (range: 0.51–0.73, bare OSLD), 0.77 (range: 0.66–0.91, jacketed OSLD), and 0.68 (range: 0.61–0.75, Eclipse). The bolusing effect of the OSLD jacket is a plausible explanation for the enhanced reading measured in that geometry. Average skin dose shows less variability under the Superflab and brass mesh boluses. The average doses as measured by EBT3 are 0.99 (range: 0.88–1.07, brass mesh), 1.02 (range: 0.98–1.08, 3 mm Superflab), 1.03 (range: 0.99–1.08, 5 mm Superflab), 1.07 (range: 1.04–1.11, 10 mm Superflab). Eclipse predicts average doses of 0.98 (range: 0.92–1.02, 3 mm Superflab), 0.98 (range: 0.91–1.03, 5 mm Superflab), and 1.04 (range: 1.03–1.06, 10 mm Superflab).

The average skin dose for 5 mm Superflab with bolus frequency of 7 out of 15 fractions is: 0.82 (range 0.75–0.89, EBT3), 0.81 (range: 0.72–0.88, bare OSLD), 0.89 (range: 0.82–0.97, jacketed OSLD), and 0.82 (range: 0.75–0.88, Eclipse).

### 3.B VMAT

**Fig. 3:**
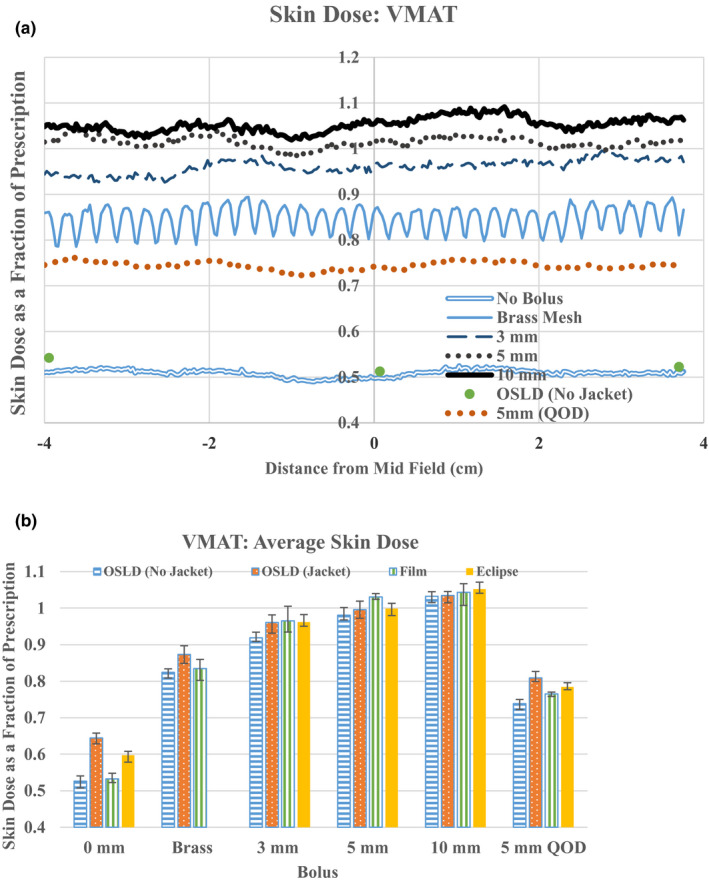
VMAT superficial doses. Skin dose measured with EBT3 where negative distance is in the lateral direction. For comparison skin doses measured by bare OSLDs are shown for the case of no bolus (a). Average skin doses in the three OLSD locations as measured by jacketed, bare OSLD, EBT3, and calculated by Eclipse (b). All doses are expressed as a fraction of prescription dose. The bars indicated the range of measurements.

In contrast to the field in field tangent plans, the doses across the films in the VMAT plans were effectively uniform as shown in Fig. 3(a). Furthermore, they are uniformly lower than the tangent plans. This is likely attributable to the greater degree of *en face* beam delivery in VMAT with its concomitant skin sparing.

We found that moving the table up by 2 mm to simulate small breathing motion or setup uncertainty resulted in decreases of superficial dose as measured by the microDiamond detector of approximately 8%, 3.5%, and 2% in the VMAT:0Bolus: −3 mmCrop plan, VMAT:10 mmBolus: 0Crop, and VMAT: 10 mmBolus: +5 mmCrop, respectively. Due to significant dependence on setup variation and breathing motion we rejected VMAT: 0Bolus: −3 mmCrop as a candidate for VMAT at this point. Skin doses measured for VMAT: 10 mmBolus:0Crop and VMAT: 10 mmBolus: +5 mmCrop were the same (to within experimental uncertainty); as such the doses reported hereafter are those obtained on VMAT: 10 mmBolus: +5 mmCrop due to its reduced sensitivity to setup variations. When this optimized plan is delivered with any bolus other than the 10 mm Superflab, the monitor units are adjusted down as explained above (5% for no bolus, brass mesh and 3 mm Superflab, 2.5% for 5 mm Superflab).

Volumetric modulated arc therapy surface doses measured by EBT3, bare OSLDs, and jacketed OSLDs for each bolus are shown in Fig. 3(b). The average superficial skin dose as measured by the three detectors again shows the largest variability for the case of no bolus: 0.53 (range: 0.52–0.55, EBT3), 0.53 (range: 0.51–0.54, bare OSLD), 0.64 (range: 0.63–0.65, jacketed OSLD), and 0.60 (range: 0.59–0.62, Eclipse). Similarly, average skin dose as measured by all three detectors shows a smaller discrepancy under the Superflab and brass mesh boluses. The average doses as measured by EBT3 are 0.83 (range: 0.82–0.85, brass), 0.96 (range: 0.95–0.97, 3 mm Superflab), 1.03 (range: 1.01–1.04, 5 mm Superflab), 1.04 (range 1.03–1.06, 10 mm Superflab). Eclipse predicts average doses of 0.96 (range: 0.94–0.97, 3 mm Superflab), 1.00 (range: 0.98–1.02, 5 mm Superflab), and 1.05 (range: 1.03–1.06, 10 mm Superflab).

### 3.C Patient data

**Fig. 4:**
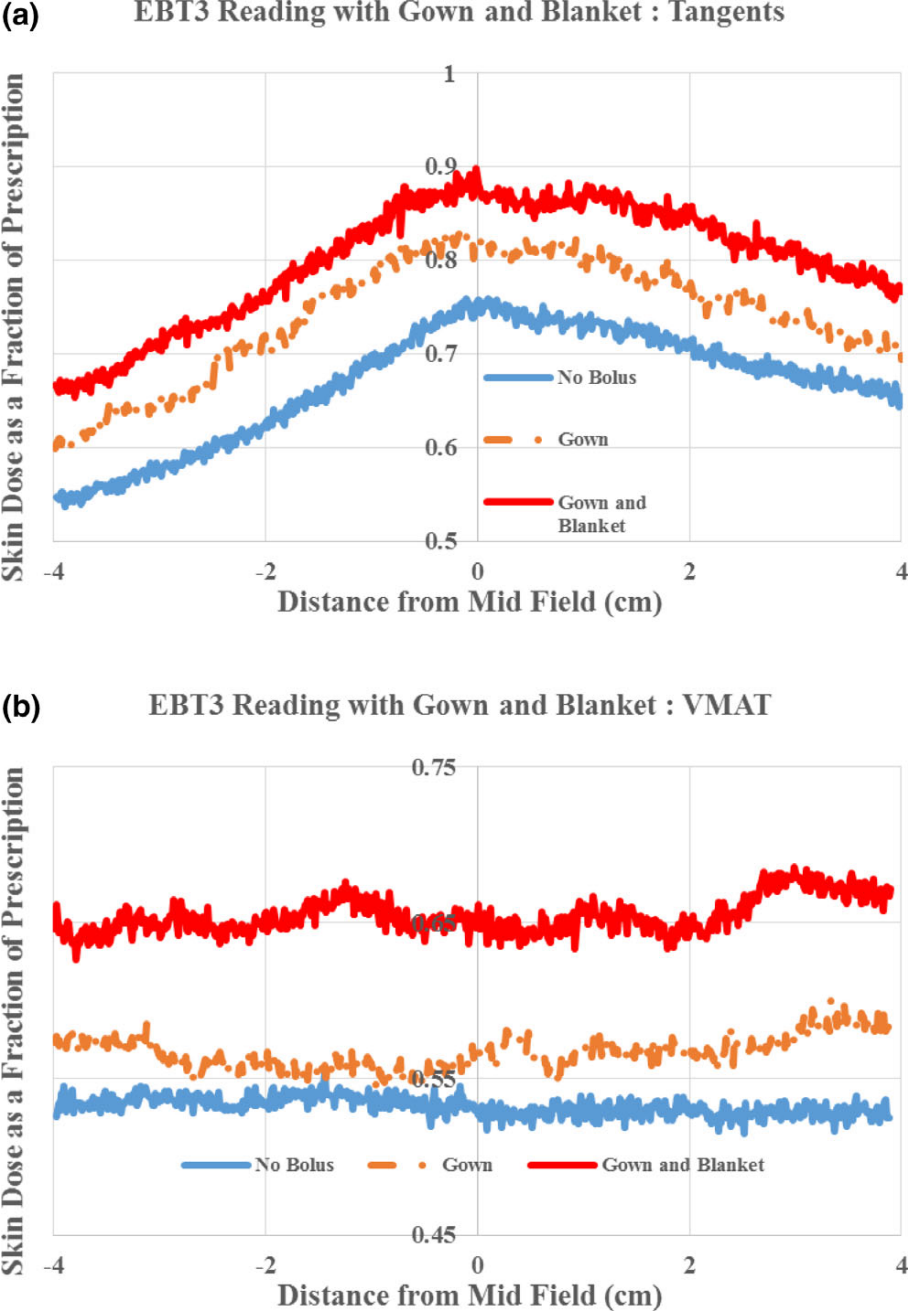
Superficial doses measured by EBT3 with no bolus in the presence of hospital gown and blanket used in our institute for tangents (a) and VMAT (b). All doses are expressed as a fraction of prescription dose.

Based on our phantom measurements we expected an average reading of 0.64 (no bolus) and 1.04 (bolus) of prescription across the OSLDs and patients. The average readings were 0.79 (range: 0.72–0.92, no bolus) and 1.0 (range 0.89–1.06, bolus). We attribute this difference mainly to the presence of gowns and hospital blankets used by our patients. All reported phantom measurements in this study were carried out without the use of any blanket, hospital gowns, or sheets. To validate our hypothesis, we performed phantom measurements in the presence of a gown and a hospital blanket.The measured no bolus VMAT values as measured by jacketed OSLDs increased to 0.81 (from 0.64). Figure 4 shows EBT3 phantom readings with no bolus for tangents (a) and VMAT (b) in the presence of hospital gown and blanket utilized in our institute.

## 4 DISCUSSION

Various authors have previously reported on the bolusing effects of different materials in post‐mastectomy radiation therapy. In this work, we have expanded on these other studies to investigate differences in bolus effects between conventional tangential beam arrangements and VMAT‐style arc deliveries. Bolus policy is often based on skin outcomes in patients receiving static, tangential beams. For this reason, we have measured the skin dose under various bolus conditions for a tangent‐based plan in this study to provide a benchmark against which we can interpret the skin dose results from the VMAT plans. Film dose measurements in the absence of bolus showed that a tangential beam arrangement will deliver a skin dose of approximately 0.64 (range: 0.54–0.73) of the prescription dose across the chest wall. The skin dose was highest at the midpoint between the medial and lateral fields and decreased toward either end. This was true for all bolus types and thicknesses as well. Interestingly, all bolus materials and thicknesses produced approximately the same dose enhancement at this midpoint (~1.07 of prescription dose), but the dose decrease at the edges was bolus dependent. The largest decrease was associated with the brass mesh (0.82 laterally, 0.96 medially), followed by 3 mm Superflab (0.92 laterally, 1 medially), 5 mm Superflab (0.97 laterally, 1.02 medially), and 10 mm Superflab (0.99 laterally, 1.05 medially). A weighted combination of the no‐bolus measurement with the 5 mm Superflab bolus (7/15 bolus fractions) revealed an average skin dose of approximately 0.82 as measured by EBT3. Manger et al.^20^ report a dose of 0.8 (EBT3, QOD 5 mm Superflab); Ordonez‐Sanz et al.^21^ report a dose of 85% (TLD‐100, QOD 10 mm Vaseline bolus).

The behavior of skin dose as a function of position along the chest wall and bolus material and thickness in VMAT plans was significantly different than in the tangents plan. Notably, the variability in skin dose as a function of position along the chest wall was markedly reduced in the VMAT plan, likely due to the less tangential weighting of the dose. The skin dose (EBT3) with no bolus in the VMAT plan was significantly lower than in the tangent plan (0.53 vs 0.64) and there was more stratification of skin dose by bolus type and thickness: brass mesh: 0.83, 3 mm Superflab: 0.96, 5 mm Superflab: 1.03, 10 mm Superflab: 1.04. In order to match 5mm QOD bolus use in tangent, these measurements suggest that brass mesh could be used on a daily basis in VMAT treatments. Alternatively, 7/15 bolus fractions would produce skin doses of 0.73, 0.76, and 0.77 for 3, 5, and 10 mm Superflab, respectively.

A single policy for bolus use, independent of treatment delivery type, would likely result in fewer treatment delivery errors. Our data indicate that perfect matching of skin doses is not possible between tangents and VMAT when a common bolus policy is used; however, differences in skin dose between the delivery techniques can be minimized. For example, implementation of a 7/15 weighting with 5 mm Superflab would produce an average skin dose (EBT3) of 0.82 with tangents and 0.76 with VMAT. Daily use of brass mesh would be well suited to VMAT deliveries but would produce unacceptably high skin doses in tangent‐based plans. Ordonez‐Sanz et al.^21^ suggest use of brass mesh in 80% of treatments in tangent‐based plans for an average skin dose of 85% as measured by TLD‐100. Healy et al.^22^ report an average dose of 100% of prescription in tangents as measured by TLD‐100 or MOSFETS under brass mesh. Also consistent with other authors’ findings,^23^, ^24^ we found the presence of flash necessary in VMAT post‐mastectomy radiotherapy.

The results of our* in vivo* study and the subsequent investigation into the bolusing effects of hospital gown and blankets, emphasize the importance of understanding the bolusing behavior of these materials. In the absence of bolus, we measured approximately 10% (of the prescription) increase in dose when a gown and blanket were both used.

Brass mesh is a convenient form of bolus since it does not need to be included in the planning process and only exerts its effect at superficial depths (i.e., little perturbation of depth dose data beyond d_max_). Our data suggest that brass mesh is roughly equivalent to the use of a 2 to 3 mm bolus in tangent treatments. However, in VMAT deliveries, there was a significant difference between these two bolus types with brass mesh producing approximately 10% (of the prescription) lower skin doses than 3 mm Superflab bolus.

The accuracy of commercial treatment planning systems (i.e., Eclipse, AAA) for the determination of skin dose has been reported in the literature.^8^, ^25^The findings of this study are in general agreement with previously published work with respect to the no bolus plans: AAA overestimated the skin dose compared to film and unjacketed OSLDs for both tangents and VMAT plans. Jacketed OSLDs measured higher doses than predicted by Eclipse, likely because of the inherent build‐up of the jacket material. Under all thicknesses of Superflab bolus, Eclipse agreed with the average of all three dosimeter measurements to within 5% of measurements for both tangents and VMAT plans, whereas with no bolus, discrepancies between measurements and Eclipse are on the order of 15%. Since we used synthetic bolus which is created in Eclipse in the planning process (as opposed to scanning the phantom with bolus), this study also validates the accuracy of dose calculation with synthetic bolus.

A potential limitation of this study is the small sample size of plans used to model post‐mastectomy radiation therapy. The printed phantom cannot be said to be representative of all post‐mastectomy radiation therapy patients and the patterns of aperture modulation in the VMAT plans could differ from those of a typical clinical plan. However, the planning techniques used for all plans in this study were the same as those used clinically for patients and the dose weighting as a function of control point in the VMAT plan was quite comparable to those typically found in clinical plans (data not shown). The conclusions drawn from the data are not intended to be absolute, but we believe that our model of dose deposition in the printed phantom is likely a reasonable approximation of generalized behavior in the post‐mastectomy radiation therapy setting. There could, of course, be examples of anatomy that would produce conclusions that differ from those presented here, but the same is necessarily true of any model of post‐mastectomy radiation therapy skin dose.

## 5 CONCLUSIONS

Clinical experience with skin dose in a post‐mastectomy radiation therapy setting has historically been derived from treatment plans that make use of tangential beam arrangements. The data in this study indicate that VMAT treatments can produce significantly different results depending on the bolus material and thickness. With the materials in this study, it was not possible to identify a single bolus policy (i.e., same material, thickness, and frequency) that produced identical skin doses; however, the use of 5 mm Superflab QOD came close, with skin doses of 0.82 of prescription dose with tangents and 0.76 with VMAT as measured by EBT3. The use of common patient garments and blankets produce bolusing effects that should not be ignored. A simple introduction of a small simulated patient motion demonstrated that incorporation of flash via PTV expansion into the bolus can significantly reduce the positional sensitivity of measured skin doses.
